# Zinc oxide nanoparticles and spironolactone-enhanced Nrf2/HO-1 pathway and inhibited Wnt/β-catenin pathway in adenine-induced nephrotoxicity in rats

**DOI:** 10.1080/13510002.2022.2139947

**Published:** 2022-11-07

**Authors:** Amira Awadalla, Eman T. Hamam, Fardous F. El-Senduny, Nisreen Mansour Omar, Mohamed R. Mahdi, Nashwa Barakat, Omar A. Ammar, Abdelaziz M. Hussein, Ahmed A. Shokeir, Salma M. Khirallah

**Affiliations:** a Center of Excellence for Genome and Cancer Research, Urology and Nephrology Center, Mansoura University, Mansoura, Egypt; b Chemistry Department, Faculty of Science, Mansoura University, Mansoura, Egypt; c Department of Pathology & Laboratory Medicine, Sylvester Comprehensive Cancer Center, Miami, FL, USA; d Department of Medical Physiology, Faculty of Medicine, Mansoura University, Mansoura, Egypt; e Department of Human Anatomy and Embryology, Faculty of Medicine, Mansoura University, Mansoura, Egypt; f Urology and Nephrology Center, Mansoura University, Mansoura, Egypt; g Basic Science Department, Delta University for Science and Technology, Gamasa, Egypt; h Biochemistry Division, Chemistry Department, Faculty of Science, Port Said University, Port Said, Egypt

**Keywords:** ZnO-NPs, spironolactone, CKD, Wnt, β-catenin, Nephrotoxicity, Nrf2, HO-1

## Abstract

**Objective:**

To investigate the renoprotective, the antioxidant, and the anti-inflammatory impact of a combination of SPL and ZnO-NPs to combat against chronic kidney disease (CKD).

**Methods:**

In total, 50 males of rats were distributed into 5 groups (10 rats each); normal group, adenine sulfate (0.25% in diet for 10 days) (CKD) group. After the last dose of adenine sulfate, rats were divided into three groups: SPL + Adenine sulfate group; rats were treated orally by mixing SPL (20 mg/kg/day) into chow for 8 weeks, ZnO-NPs + Adenine sulfate group; rats were injected intraperitoneally with ZnO-NPs (5 mg/kg) three times weekly for 8 weeks, ZnO-NPs + SPL + Adenine sulfate group; rats were injected with the same previous doses for 8 weeks.

**Results:**

Each of SPL and ZnO-NPs up-regulated antioxidant genes (*Nrf2* and *HO-1*), down-regulated fibrotic and inflammatory genes (*TGF-β1, Wnt7a, β-catenin, fibronectin, collagen IV, α-SMA*, *TNF-α,* and *IL-6*) compared to CKD. Furthermore, a combination of SPL and ZnO-NPs resulted in a greater improvement in the measured parameters than a single treatment.

**Conclusion:**

The therapeutic role of SPL was enhanced by the antioxidant and the anti-inflammatory role of ZnO-NPs, which presented a great renoprotective effect against CKD.

## Introduction

Chronic kidney disease (CKD) is a severe public health disease that causes irreversible destruction of kidney and eventually leads to tubulointerstitial fibrosis and glomerular damage, which is the main pathway to end-stage renal disease (ESRD) [1].

CKD causes an increase in oxidative stress, which causes inflammation, renal damage, and death of cells through NF-κB activation and Nrf2/HO-1 inhibition in renal tissues [2]. Moreover, CKD activates Wnt/β-catenin pathway, resulting in the elevation of oxidative stress and renal fibrosis [3].

Currently, the use of zinc oxide nanoparticles (ZnO-NPs) has become essential due to their ability to pass through cell membranes. Zinc was reported as a pro-antioxidant agent due to its role in the protection of thiol-containing proteins (antioxidant enzymes) and zinc-finger transcription factors [4]. In rats, ZnO-NPs protect against acute kidney injury (AKI) caused by cisplatin [5]. Furthermore, ZnO-NPs alleviate diabetic nephropathy, the main cause of ESRD [6]. Moreover, ZnO-NPs are dietary supplements with anti-inflammatory properties revealed by down-regulating mRNA expressions of *IL-1β, IL-6,* and *TNF-α*. ZnO-NPs are approved as anticancer therapies due to the electrostatic attraction that occurs between negatively charged cancer cells and positively charged ions of ZnO-NPs, inducing apoptosis. Furthermore, ZnO-NPs were conjugated with anti-diabetic drugs to improve their effect due to their antioxidant activity [5].

In addition, Spironolactone (SPL) is a synthetic aldosterone antagonist and was approved as a diuretic medicine for treating hypertension, primary hyperaldosteronism, and edematous states. Therapeutic indications of SPL were subsequently expanded in response to mounting evidence of the systemic pro-inflammatory and pro-fibrotic effects, mainly in the heart, kidneys, and vessels. In CKD, aldosterone levels typically rise as the glomerular filtration rate (GFR) decreases, so both ESRD and CKD reflect relative hyperaldosteronism. Several clinical trials have shown that SPL has a good nephroprotective effect [7]. SPL inhibited profibrotic effects of TGF-β1, preventing fibrosis in kidney tissue, and promoting cellular recovery [8].

Previous research has shown that ZnO-NPs have renoprotective effects against AKI [5] and CKD [9] in rats. Human and rat studies have also demonstrated that SPL can combat against CKD [7,10]. Therefore, this study investigated the impact of this combination in comparison to the effect of each agent alone in the renoprotection, antioxidant, and anti-inflammation against CKD induced by adenine sulfate in rats. Moreover, we aimed to study the mechanisms by which this combination works.

## Materials and methods

### Experimental groups

Fifty mature males of Sprague–Dawley rats weighing 180 ± 200 g were divided at a rate of four rats per polycarbonate cage. They were kept at 24°C with 50–70% humidity and 12 h dark light cycle. All procedures used in this study were approved by Institutional Animal Ethics Committee of Faculty of Medicine, Mansoura University, Egypt [R.22.03.1638]. Rats were distributed into five equal groups (10 rats each); (a) normal healthy (control) group, (b) adenine sulfate (CKD positive control) group; for 10 days, the diet included 150 mg/kg/day (0.25% w/w) powdered adenine sulfate [11], (c) SPL + adenine sulfate group; adenine sulfate group was treated orally for eight weeks by mixing SPL (20 mg/kg/day) into chow [10], (d) ZnO-NPs + adenine sulfate group; adenine sulfate group injected intraperitoneal (i.p)with ZnO-NPs (5 mg/kg) three times weekly for eight weeks after the last dose of adenine sulfate [9], (e) ZnO-NPs + SPL + adenine sulfate group; adenine sulfate group treated with the same previous doses of ZnO-NPs and SPL for eight weeks after the last adenine sulfate dose.

### Samples collection

Every rat was sited in a metabolic cage for 24 h to collect urine and blood samples from the heart before the sacrifice of the animal under inhalational general anesthesia by sodium thiopental. For biochemical measurements, samples of blood were centrifuged, and serum was collected and stored at −20°C. Then, cervical dislocation was used to sacrifice the animals, and a midline laparotomy and bilateral nephrectomy were performed. The right kidney was fixed in 10% buffered formalin for immunohistochemical and histopathological studies, whereas the left kidney was kept at −80°C till biochemical and molecular parameters were performed.

### Measurements of kidney functions

Serum and urine creatinine (creatinine clearance (CrCl)), serum blood urea nitrogen (BUN), and microalbuminuria were colorimetrically measured according to manufacturer guidelines [12,13]. Kits were provided by Diamond Diagnostics, Egypt.

### Evaluation of antioxidant and oxidative stress parameters

A colorimetric method was used to measure catalase (CAT), superoxide dismutase (SOD), reduced glutathione (GSH), and malondialdehyde (MDA) in the supernatant of kidney homogenates [12]. Kits were provided by Biodiagnostic, Egypt.

### Gene expression investigation

Total renal RNAs were isolated using TRIzol (Invitrogen). mRNAs expression levels were measured using StepOne plus by QuantiFast SYBR Green PCR Kit (Qiagen, Germany). Fibrotic genes (*TGF-β1, Wnt7a, β-catenin, fibronectin, collagen IV,* and *α-SMA*), inflammatory genes (*IL-6* and *TNF-α*), and antioxidants genes (*Nrf2* and*HO-1*) were evaluated by real-time PCR. As shown in Table 1, primers were created online at NCBI and manufactured at Vivantis (Malaysia). Gene transcription was normalized to GAPDH. The mRNA expression level was calculated by the 2^-ΔΔct^ Equation [14].

**Table 1.** PCR primers.

Common name	Sequence (5′−3′)	Accession no.
*TGF-β1*	F: 5′-CACTCCCGTGGCTTCTAGTG-3′ R: 5′-GGACTGGCGAGCCTTAGTTT-3′	NM_021578.2
*Wnt7a*	F: 5′-GCCCACCTTTCTGAAGATCAAG-3′ R: 5′-TGGGTCCTCTTCACAGTAATTGG-3′	NM_001100473.1
*β-catenin*	F: 5′-ACAGCACCTTCAGCACTCT-3′ R: 5′-AAGTTCTTGGCTATTACGACA-3'	NM_053357.2
*Fibronectin*	F: 5′-GTGGCTGCCTTCAACTTCTC-3′ R: 5′-GTGGGTTGCAAACCTTCAAT-3′	NM_019143.2
*Collagen IV*	F: 5′-ATAGAGAGAAGCGAGATGTTCAAGA-3′ R: 5′-GGATATAATTCTAGGGTTCGTTGCT-3′	NM_001135009.1
*α-SMA*	F: 5′-TTCGTGACTACTGCTGAGCG-3′ R: 5′-AAGCGTTCATTCCCGATGGT-3′	NM_031004.2
*TNF-α*	F: 5′-TTCGGAACTCACTGGATCCC-3′ R: 5′-GGAACAGTCTGGGAAGCTCT-3′	NM_012675.3
*IL-6*	F: 5′-GCCCTTCAGGAACAGCTATGA-3′ R: 5′-TGTCAACAACATCAGTCCCAAGA-3′	NM_012589.2
*Nrf2*	F: 5′-ATTGCTGTCCATCTCTGTCAG-3′ R: 5′-GCTATTTTCCATTCCCGAGTTAC-3′	NM_001399173.1
*HO-1*	F: 5′-TGCTTGTTTCGCTCTATCTCC-3′ R: 5′-CTTTCAGAAGGGTCAGGTGTC-3′	NM_012580.2
*GAPDH*	F: 5′-TATCGGACGCCTGGTTAC-3′ R: 5′-CTGTGCCGTTGAACTTGC-3′	NM_017008.4

### Western blot analysis

The nuclear and cytoplasmic fractions were prepared by using the sucrose gradient protocol [15]. The concentration of protein was measured by Pierce™ BCA Protein Assay Kit. A 30 µg/well was loaded to SDS-PAGE. Then, the protein was transferred to 0.45 µm nitrocellulose membrane for 90 min at 90 V. The protein transfer was confirmed using Ponceau S stain. The membrane was blocked for 2 h with 3% BSA at room temperature and then incubated with primary antibodies against Nrf2, β-Catenin, PCNA (Proliferating cell nuclear antigen) (Thermo Scientific, MS-106) or actin (Cell signaling technology) at 4°C overnight. After washing the membrane, it was incubated with anti-rabbit HRP-conjugated secondary antibody for one hour at room temperature. After washing the membrane, the signal was detected using WesternBright™ ECL HRP substrate (Advansta, K-12045) and visualized by The ChemiDoc MP Imaging System (Bio-Rad). The fold of change in protein level was calculated using GraphPad Prism Software after normalization to the level of housekeeping protein β-actin in cytoplasmic fraction or PCNA in the nuclear fraction.

### Histopathological analysis

The kidney was dehydrated by the serial ascending concentration of alcohol (BDH, UK), and xylene (BDH, UK), then the tissues were embedded in paraffin wax (Sherwood, USA). The block of paraffin embedding tissue was cut at 5 µm by microtome (West Germany). Haematoxylin, and eosin were used to stain the slides to investigate the tissue under the light microscope (400×) [16]. Tubulointerstitial damage, chronicity, and regeneration were evaluated using a semi-quantitative pathological score according to Shi et al*.* [17].

### Immunohistochemical investigation of β-catenin, TGF-β1, and α-SMA

Immunohistochemical investigation for kidney tissue was done to determine fibrotic markers expression (β-catenin, TGF-β1, and α-SMA) using staining by their antibodies. Slides were examined under light microscope at 200× magnification in order to detect immune reactive cells [16]. The expression of β-catenin, TGF-β1, and α-SMA (the number of positive (brown) cells) were calculated by a semi-quantitative score [13].

### Scoring of expression of beta catenin, TGF-β1, and α-SMA

The number of renal tubules stained with each marker was counted in each field (HPF) and the mean was calculated to indicate the tubular staining, and number of glomerular cells stained was counted in each glomeruli at HPF and the mean was calculated.

### Immunofluorescence examination

Immunofluorescence staining for kidney tissue was used to show collagen III and IV expression. The samples were treated with the primary antibody at 4°C overnight (1:200). After being rinsed in PBS, the samples were incubated for one hour at 25°C with a secondary antibody, anti-rat IgG (1:400). The expression of collagen III and IV among the study groups was assessed using digital images after tissue sections were examined using fluorescence microscopy [18].

### Statistical analysis

SPSS V 22 was used to conduct the statistical analysis. The data were presented as a mean ± standard deviation. The differences between groups were investigated using a one-way ANOVA analysis of variance with a post-hoc comparison. The Tukey post-test was employed to evaluate whether there were any differences between the groups. *P* value of <0.05 is considered significant. The Mann–Whitney and Kruskal–Wallis tests were used for statistical analysis of pathological score of histopathological examination.

## Results

### Effect of ZnO-NP and SPL on renal function and antioxidants activity

When compared to the normal group, the adenine (CKD) group revealed that levels of microalbuminuria, serum BUN, as well as creatinine were all significantly elevated, while creatinine clearance(CrCl) was significantly decreased (*p* < 0.05). Conversely, each of ZnO-NPs and SPL-treated groups indicated a significant reduction in microalbuminuria, serum BUN, and creatinine and a significant increase in CrCl when compared to adenine group (*p* < 0.05). However, when compared to SPL group, ZnO-NPs group showed a significant decrease in microalbuminuria, serum BUN, and creatinine levels. Furthermore, ZnO-NPs + SPL group exposed more significant attenuation in microalbuminuria, serum BUN, and creatinine levels and a significant rise in CrCl when compared to each of ZnO-NPs and SPL groups (Table 2). In comparison to the normal group, the adenine (CKD) group showed a significant rise in MDA and a decrease in SOD, GSH, and CAT levels (*p* < 0.05). Interestingly, MDA was significantly reduced while GSH, SOD, and CAT were considerably higher in each of ZnO-NPs and SPL-treated groups when compared to CKD group (*p* < 0.05). However, as compared to SPL group, there was no significant difference in oxidative stress or antioxidants in ZnO-NPs group. Furthermore, when compared to each of SPL and ZnO-NPs groups, the SPL + ZnO-NPs group had a greater reduction in MDA and a greater improvement in SOD, GSH, and CAT levels (Table 3).

**Table 2.** Treatment of CKD rat model with ZnO-NP and SPL results in improvement of renal function.

Variables (mean ± SD)	Control	CKD	SPL	ZnO-NPs	SPL + ZnO-NPs
Serum creatinine (mg/dL)	0.68 ± 0.19	4.09 ± 0.9^a^	2.74 ± 0.29^ab^	1.53 ± 0.36^abc^	0.8 ± 0.16^bc^
BUN (mg/dL)	18.99 ± 1.95	54.36 ± 6.99^a^	41.98 ± 6.73^ab^	32 ± 7.05^abc^	21.4 ± 2.33^abcd^
Crcl (mL/min)	1.44 ± 0.16	0.54 ± 0.17^a^	0.83 ± 0.09^ab^	0.93 ± 0.11^ab^	1.16 ± 0.12^abcd^
Microalbuminuria (mg/24 h)	20.69 ± 1.6	96.8 ± 13.22^a^	68.9 ± 10.2^ab^	52.55 ± 7.74^abc^	35.54 ± 5.45^abcd^

Notes: Where control group: normal group, CKD group: adenine sulfate (positive control group), SPL group: SPL + adenine sulfate, ZnO-NPs group: ZnO-NPs + adenine sulfate, SPL + ZnO-NPs group: SPL + ZnO-NPs + adenine sulfate.

Significant difference compared to corresponding ^a^control group, ^b^CKD group,^c^SPL group, ^d^ZnO-NPs group at *p* < 0.05 by ANOVA and Tukey test.

**Table 3.** Treatment of CKD rat model with ZnO-NP and SPL results in down-regulation of oxidative stress and enhanced antioxidants.

Groups	Control	CKD	SPL	ZnO-NPs	SPL + ZnO-NPs
GSH (mmol/g)	9.4 ± 2.5	2.5 ± 0.73^a^	4.44 ± 1.41^a^	5.78 ± 1.14^ab^	8.39 ± 1.72^bc^
SOD (U/g)	324.7 ± 41.21	121.6 ± 37.2^a^	217.03 ± 34.7^ab^	260.14 ± 33.8^ab^	297.6 ± 21.27^bc^
CAT (U/g)	8 ± 3.32	1.68 ± 0.51^a^	5.25 ± 1.32^ab^	6.03 ± 1.21^ab^	7.36 ± 2.11^b^
MDA (nmol/g)	11.58 ± 3.33	49.77 ± 11.05^a^	32.37 ± 5.96^ab^	24 ± 5.65^ab^	15.9 ± 4.12^bc^

Notes: Significant difference compared to corresponding ^a^control group, ^b^CKD group, ^c^SPL group, ^d^ZnO-NPs group at *p* < 0.05 by ANOVA and Tukey test.

### Effect of ZnO-NP and SPL on fibrotic, inflammatory, and antioxidant gene expression

**Figure 1.**
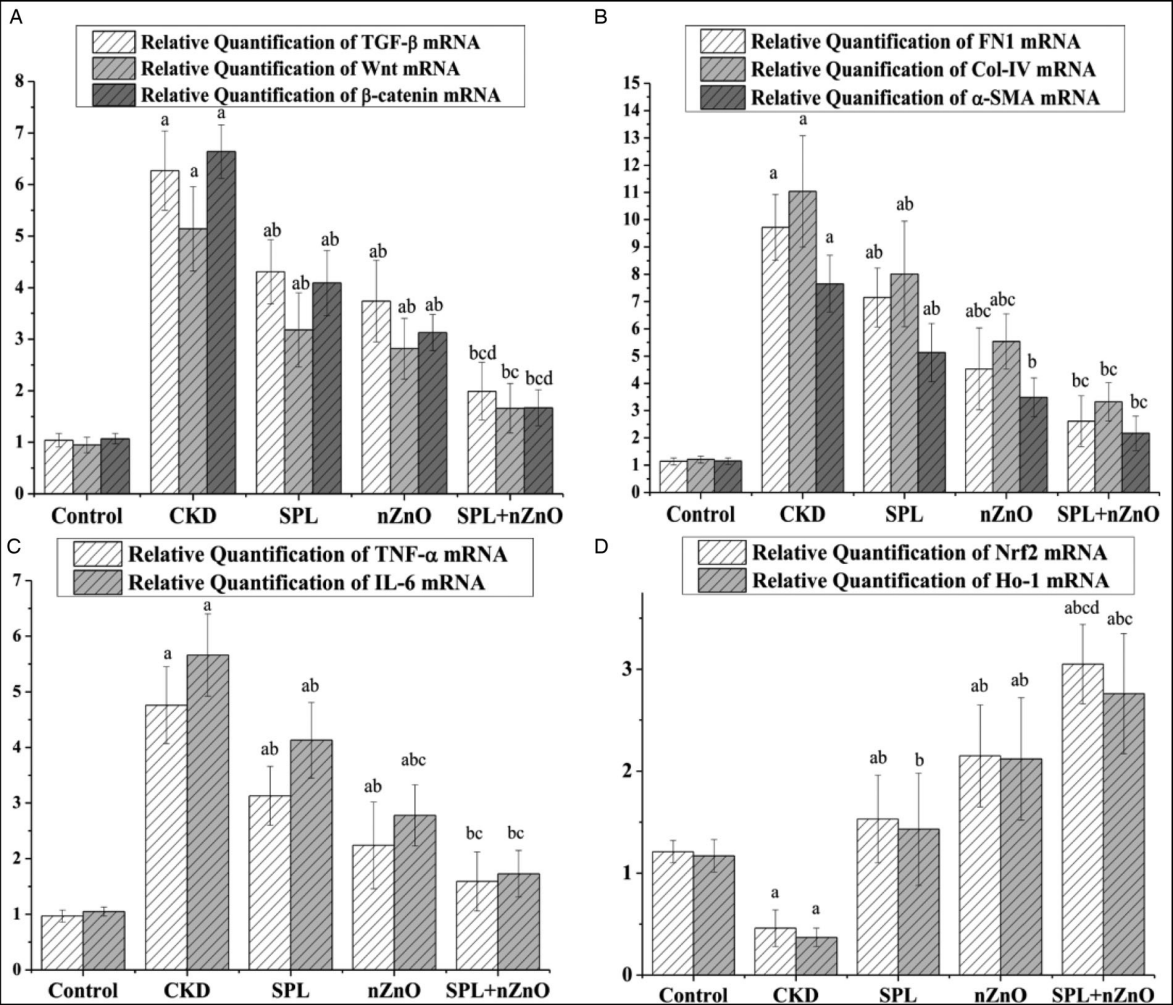
Treatment of CKD rat model with ZnO-NP and SPL results in down-regulation of fibrotic and inflammatory gene expression and enhanced antioxidant gene expression. Significant difference compared to corresponding ^a^control group, ^b^CKD group, ^c^SPL group, ^d^ZnO-NPs group at *p* < 0.05 by ANOVA and Tukey test. Regarding levels of relative quantification of TGF-β1 mRNA, *Wnt7a* mRNA, and β-catenin mRNA (A), relative quantification of FN1 mRNA, Col-IV mRNA, and α-SMA mRNA (B), relative quantification of TNF-α mRNA and IL-6 mRNA (C), and relative quantification of Nrf2 mRNA and HO-1 mRNA (D).

When compared to the normal group, fibrotic genes (*TGF-β1*, *Wnt7a*, *β-catenin*, *fibronectin*, *collagen IV*, and *α-SMA*), and inflammatory genes (*TNF-α* and *IL-6*) were significantly up-regulated, while antioxidant genes (*Nrf2* and *HO-1*) were significantly down-regulated in kidney tissues of adenine (CKD) group (*p* < 0.05). Conversely, when compared to CKD group, each of SPL and ZnO-NPs groups significantly down-regulated the fibrotic genes, and the inflammatory genes. Additionally, each of SPL and ZnO-NPs groups significantly up-regulated the antioxidant genes (*p* < 0.05). As compared to the SPL group, *collagen IV* and *IL-6* expression were significantly lower in ZnO-NPs group. However, ZnO-NPs and SPL group demonstrated greater attenuation of fibrotic and inflammatory genes and a more significant rise in antioxidant genes when compared to each of ZnO-NPs and SPL groups (Figure 1).

### Effect of ZnO-NP and SPL on β-catenin and Nrf2 protein expression

**Figure 2.**
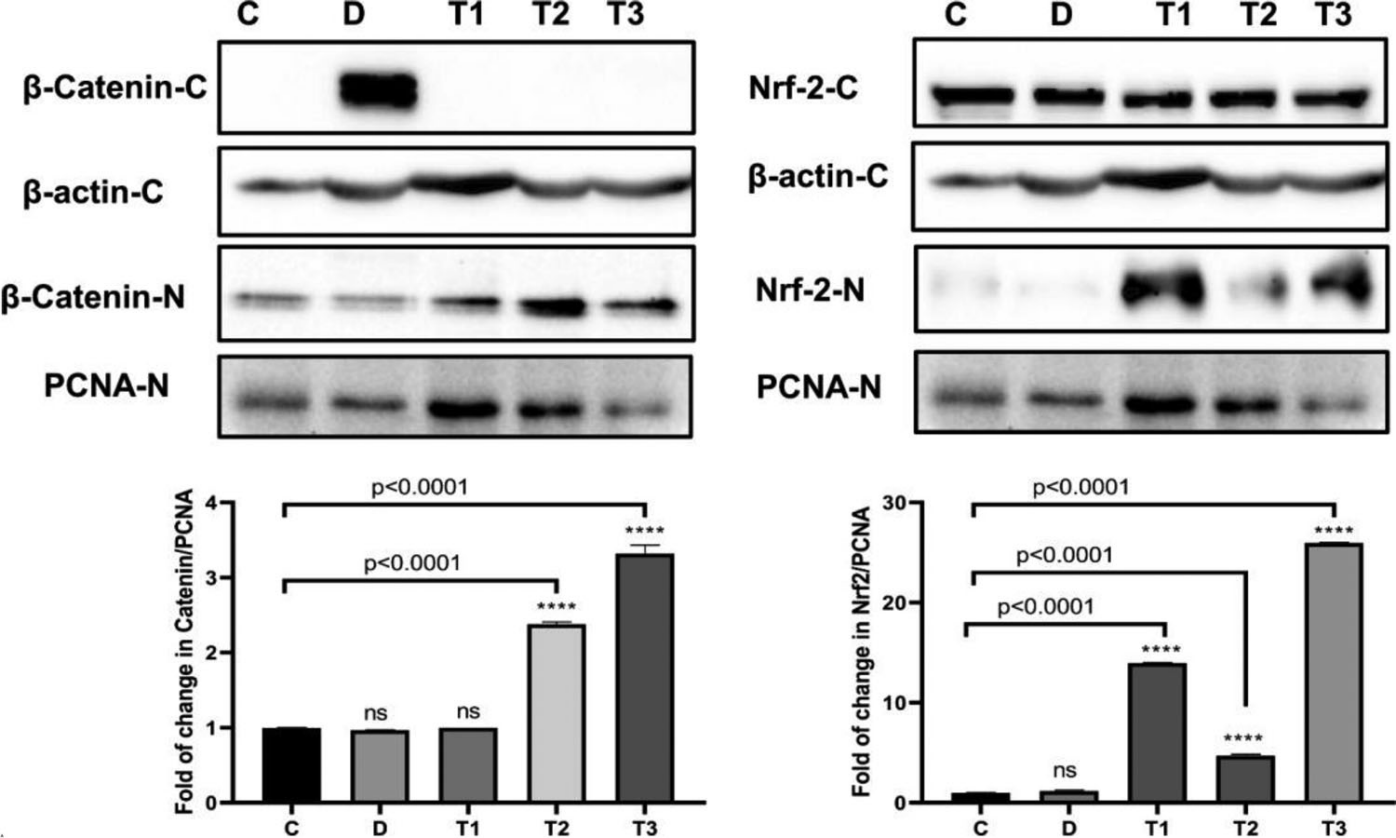
Treatment of CKD rat model with ZnO-NP and SPL results in down-regulation of β-cateninin cytoplasm and up-regulation of Nrf2 in western blot analysis. Significant difference compared to *control group at *p* < 0.0001 where C: normal control group, D: CKD group, T1: SPL group, T2: ZnO-NPs group, T3: SPL + ZnO-NPs group.

When compared to adenine (CKD) group, western blot analysis after normalization to the cytoplasmic actin or nuclear PCNA protein revealed that in each of ZnO-NPs and SPL + ZnO-NPs groups, β-catenin was decreased in cytoplasm and significantly translocated to the nucleus (*p* < 0.001). Additionally, when compared to CKD group, Nrf2 nuclear translocation was significantly greater in each of ZnO-NPs and SPL + ZnO-NPs groups (*p* < 0.0001) (Figure 2).

### Histopathological, immunohistochemical, and immunofluorescence observations

**Figure 3.**
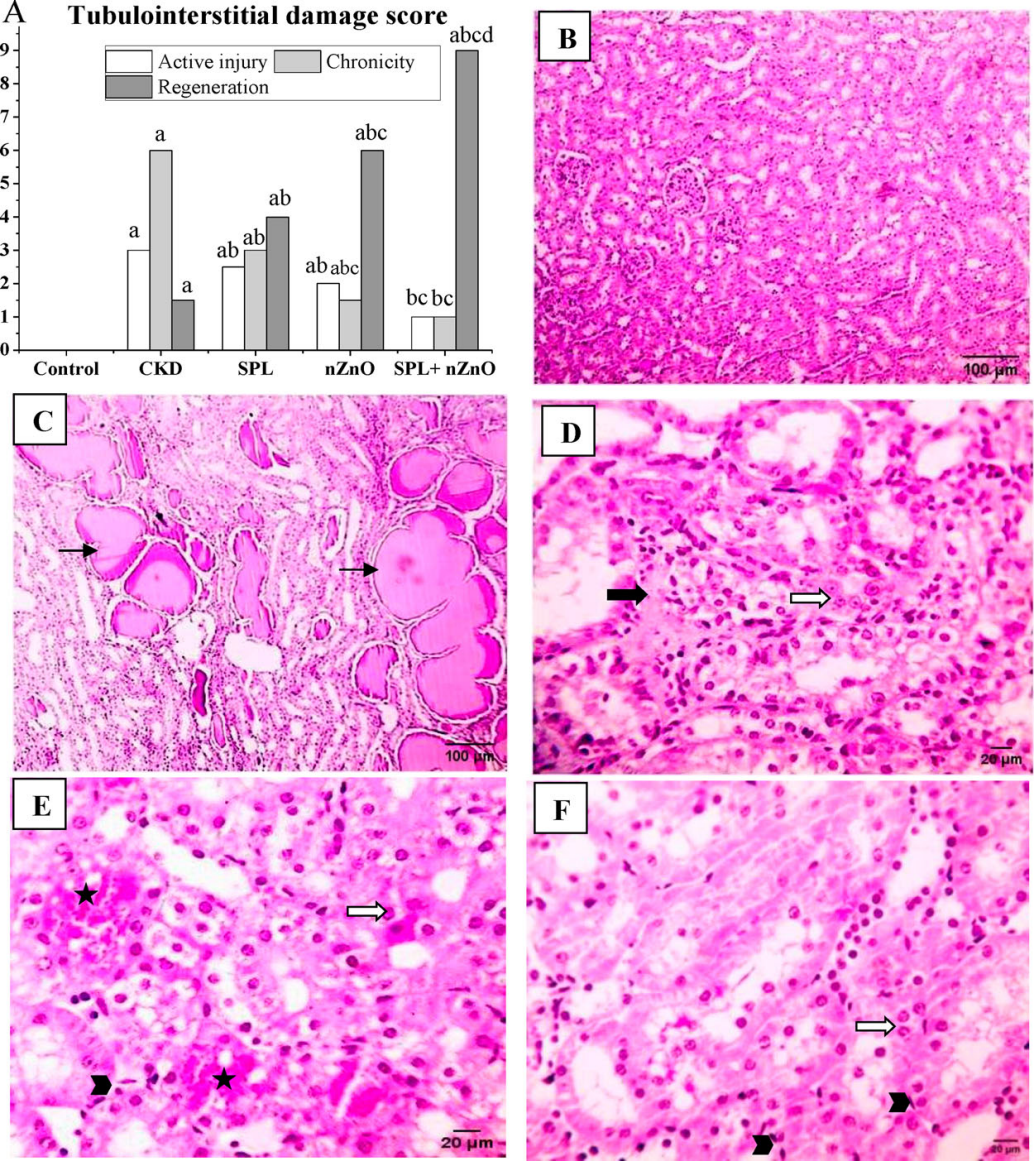
Histopathological examination of the kidney tissues with H&E. (A) The score of tubulointerstitial damage. Microscopic pictures of kidney specimens showing: (B) normal glomerular and tubular kidney structure (normal group, 100×), (C) protein leakage in the tubular lumen (CKD group, 100×), (D) mild interstitial collagen proliferation (bold black arrow) and prominent nuclei (white arrow) (SPL group, 400×), (E) mild congestion (star), mitotic figure (arrowhead) and prominent nuclei (white arrow) (ZnO-NPs group, 400×) and (F) prominent nuclei (white arrow) and mitotic figure (arrowhead) (SPL + ZnO-NPs group, 400×). a: significant difference versus control, b: significant versus CKD, c: significant versus SPL, d: significant versus ZnO-NPs at *p* < 0.05.

When compared to the adenine group, the tubulointerstitial damage score of kidney tissues was substantially higher in the adenine (CKD) group (*p* < 0.05) and dramatically enhanced in all treatment groups, especially the SPL + ZnO-NPs group. (Figure 3(A)). Normal kidney structure was seen in the normal group (Figure 3(B)), while CKD group presented protein leakage in the tubular lumen (Figure 3(C)). On the other hand, SPL group indicated mild interstitial collagen proliferation and prominent nuclei (Figure 3(D)), and ZnO-NPs group revealed mild congestion, mitotic figure and prominent nuclei (Figure 3(E)). ZnO-NPs group represented more regeneration than SPL group. Furthermore, SPL + ZnO-NPs group exposed prominent nuclei and mitotic figure (Figure 3(F)).

**Figure 4.**
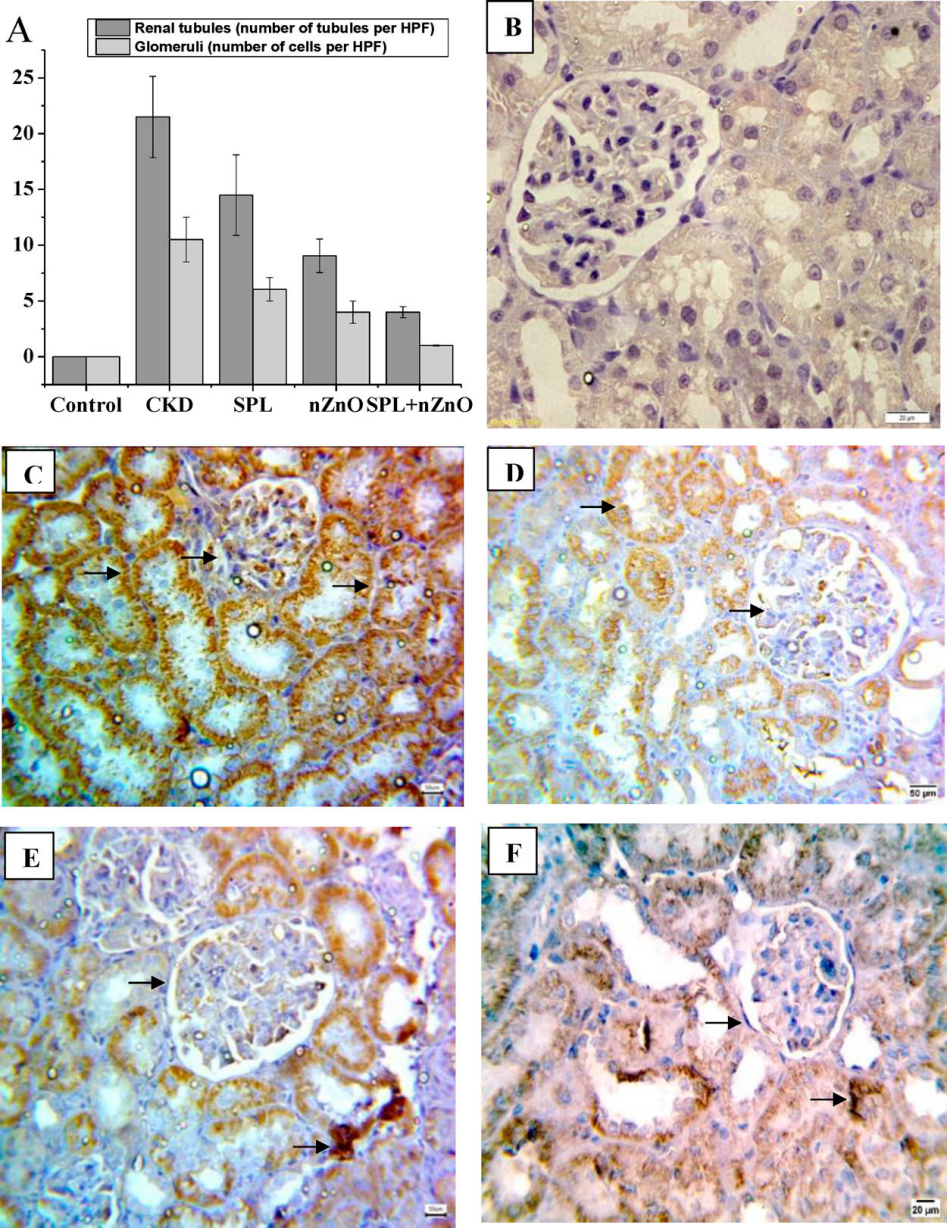
Immunohistopathological examination for β-catenin expression in kidney tissues. (A) The score of β-catenin in all studied groups, (B) negative expression for beta catenin (control group, 400×), (C) marked cytoplasmic expression for beta catenin in both renal tubules and glomeruli (CKD group, 200×), (D) moderate expression of β-catenin in SPL group (200×), (E) mild expression of β-catenin in ZnO-NPs group (200×), and (F) mild expression in SPL + ZnO-NPs group (200×). a: significant difference versus control, b: significant versus CKD, c: significant versus SPL, d: significant versus ZnO-NPs at *p* < 0.05.

**Figure 5.**
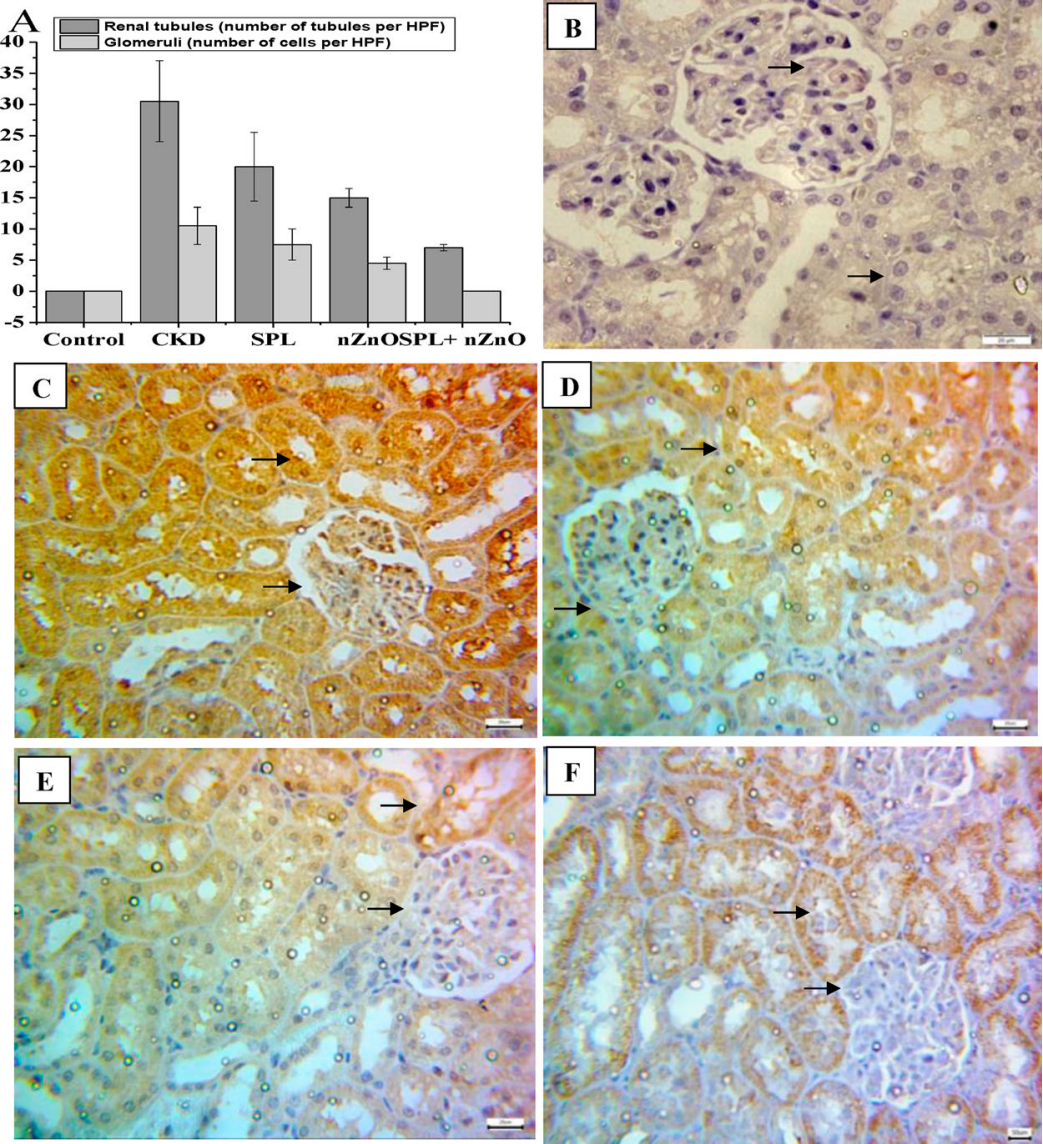
Immunohistopathological examination for TGF-β1 expression in kidney tissues. (A) The score of TGF-β1 in all studied groups, (B) negative expression for TGF-β1 (control group, 400×), (C) marked cytoplasmic expression for TGF-β1 in both renal tubules and glomeruli (CKD group, 200×), (D) moderate expression of TGF-β1 in SPL group (200×), (E) mild expression of TGF-β1 in ZnO-NPs group (200×), and (F) mild expression in SPL + ZnO-NPs group (200×). a: significant difference versus control, b: significant versus CKD, c: significant versus SPL, d: significant versus ZnO-NPs at *p* < 0.05.

**Figure 6.**
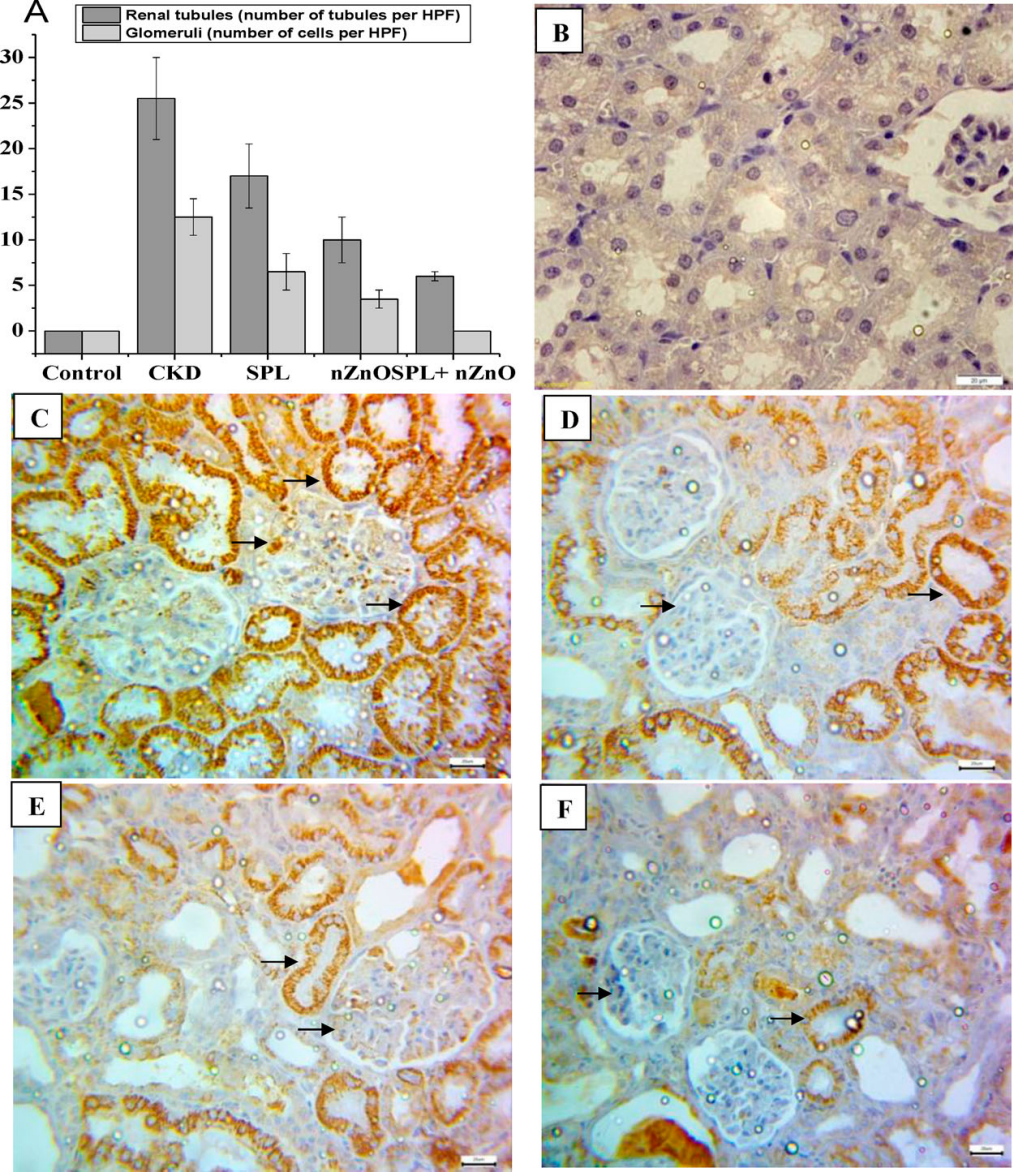
Immunohistopathological examination for α-SMA expression in kidney tissues. (A) The score of α-SMA in all studied groups, (B) negative expression for α-SMA (control group, 400×), (C) marked cytoplasmic expression for α-SMA in both renal tubules and glomeruli (CKD group, 200×), (D) moderate expression of α-SMA in SPL group (200×), (E) mild expression of α-SMA in ZnO-NPs group (200×), and (F) mild expression in SPL + ZnO-NPs group (200×). a: significant difference versus control, b: significant versus CKD, c: significant versus SPL, d: significant versus ZnO-NPs at *p* < 0.05.

As shown in Figures 4–6(A), the immunohistochemical positive scoring manifested a significant increase in β-catenin, TGF-β1, and α-SMA expression in the tubular epithelial cells and glomerular cells in CKD group as compared to the normal group. However, their expressions were considerably inhibited in all treated groups, especially SPL + ZnO-NPs group, when compared to CKD group (*p* < 0.05). Figures 4–6(B–F) depicted the changes in β-catenin, TGF-β1, and α-SMA tubular and glomerular staining among the different treated groups. Marked expression of the studied proteins was observed in CKD, while moderate and mild expressions were detected in the SPL, ZnO-NPs, and combination group, respectively.

**Figure 7.**
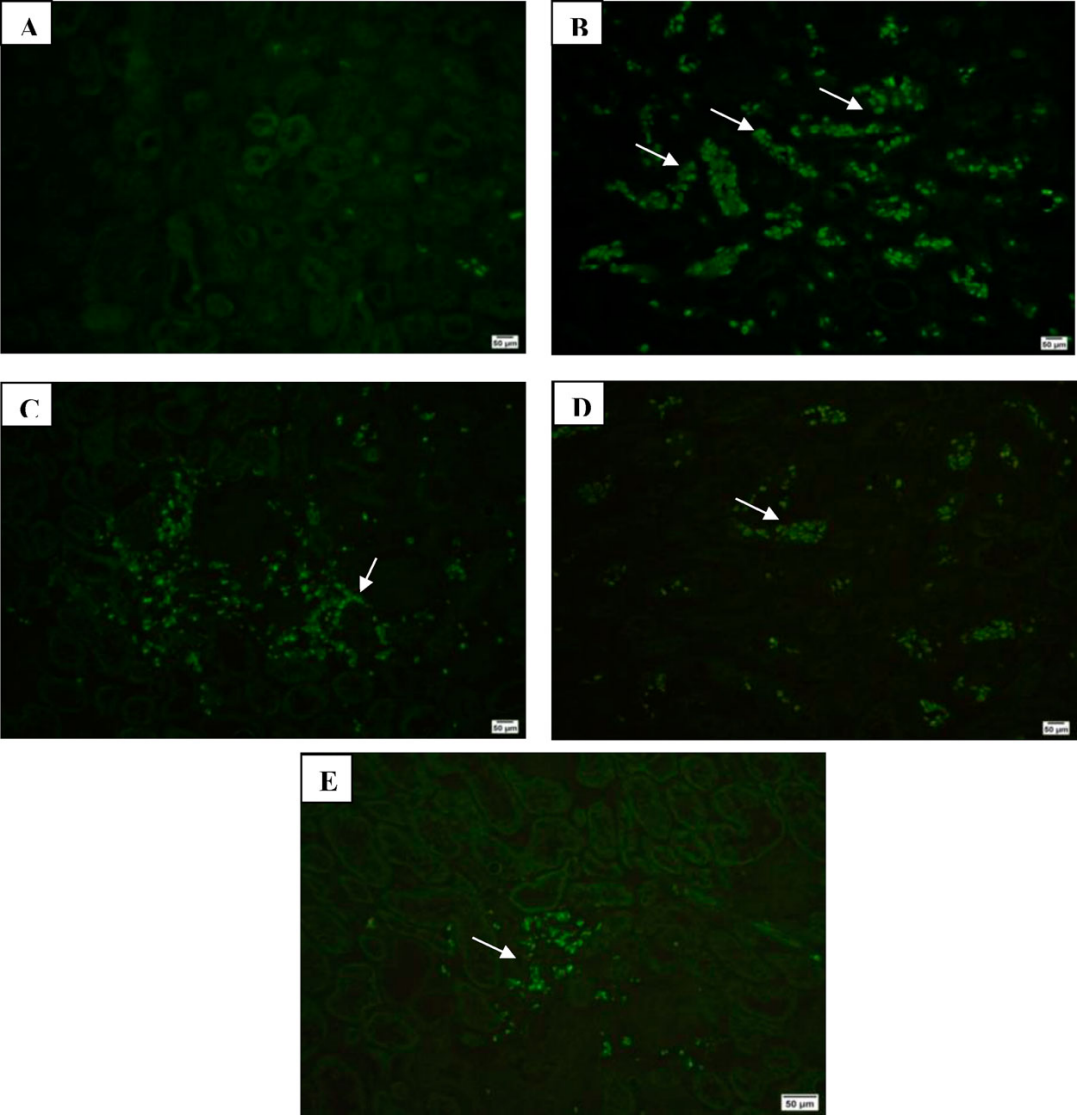
Immunofluorescence investigation of collagen III in kidney tissues showing: (A) mild expression in control group (200×), (B) marked expression in CKD group (200×), (C) moderate expression in SPL group (200×), (D) mild expression in ZnO-NPs group (200×), and (E) mild expression in SPL + ZnO-NPs group (200×).

**Figure 8.**
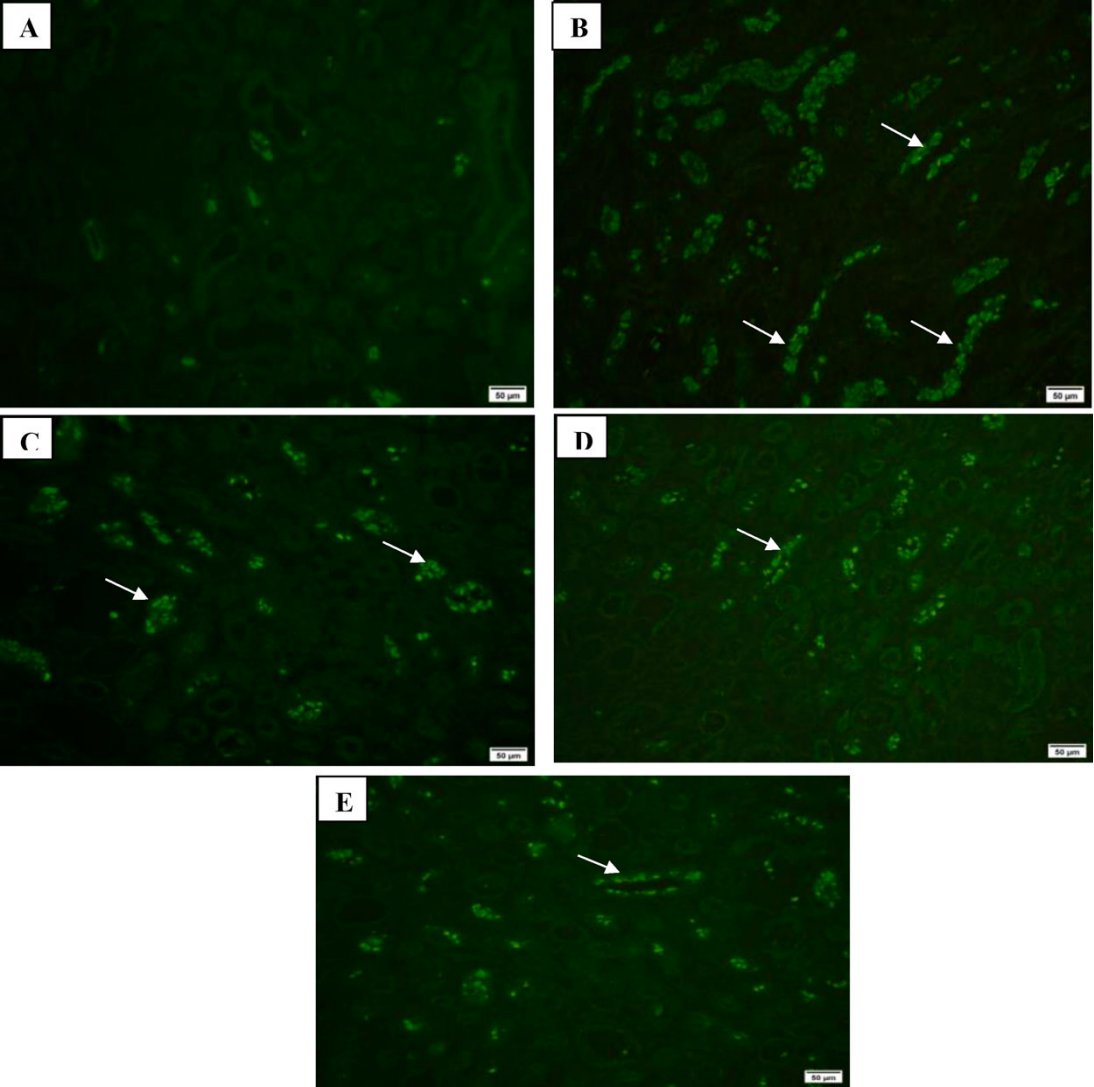
Immunofluorescence investigation of collagen IV in kidney tissues showing: (A) mild expression in control group (200×), (B) marked expression in CKD group (200×), (C) moderate expression in SPL group (200×), (D) mild expression in ZnO-NPs group (200×), and (E) mild expression in SPL + ZnO-NPs group (200×).

Additionally, Immunofluorescence examination of renal extracellular matrix proteins collagen III and IV showed marked expression in CKD group, moderate expression in SPL group and mild expression in ZnO-NPs and SPL + ZnO-NP groups (Figures 7 and 8).

## Discussion

Kidney possesses an intrinsic regeneration capacity; this regeneration is limited under chronic condition of kidney disease and cannot prevent the fibrosis process [1]. Chronic inflammation is common in CKD, as are significantly compromised anti-oxidative mechanisms. Inflammation and oxidative stress are important mechanisms of defense, but if they are not adequately managed, they can cause a variety of negative effects [19]. The use of ZnO-NPs is widespread in a variety of applications due to their distinct physical and chemical properties that allow them to interact with cellular macromolecules, resulting in a variety of therapeutic effects. Furthermore, ZnO-NPs have a wide range of biomedical applications, including antioxidant, antibacterial, anticancer, and anti-inflammatory [6]. SPL and its pharmacological properties as a diuretic drug, as well as its antioxidant and renoprotective effects, have been the subject of several studies [7]. The objective of this research was to investigate if a combination of SPL and ZnO-NPs may protect against CKD in comparison to each of the agents alone.

The adenine addition to diets of rats has become widely accepted as a model for studying kidney injury. Previous research has supported our findings on the significant declines in kidney functions and significant damage of kidney morphology [11]. Conversely, SPL and ZnO-NPs treatment improved kidney function compared to each of SPL and ZnO-NPs groups, indicating that SPL and ZnO-NPs are renoprotective against adenine-induced CKD. Furthermore, the development in histopathological results of SPL and ZnO-NPs group confirmed this result. Barakat et al. [5] showed that ZnO-NPs improved the decrease in kidney function caused by cisplatin in rats. Also, Elseweidy et al. [8] explained that SPL reversed the adenine effect through kidney function improvement, histopathological findings, and fibrosis recovery enhancement. This could be explained by the antagonistic action of aldosterone by SPL where the renin–angiotensin aldosterone system (RAAS) is considered as the major hormonal circuit that regulates blood pressure by maintaining the level of sodium and potassium [20].

The current study revealed that the oxidative stress increased and antioxidant decreased in adenine group. Previous research found that adenine feeding caused a significant increase in MDA while decreasing GSH, SOD, and CAT levels in renal tissues [21]. Furthermore, ZnO-NPs and SPL group indicated a great rise in GSH, SOD, and CAT, and a significant inhibition in MDA in renal tissues compared to each of SPL and ZnO-NPs groups. Pessôa et al. [22] confirmed that SPL increased antioxidants and reduced oxidative stress due to inflammation reduction. In addition, Abd El-Khalik et al. [6] revealed that ZnO-NPs cause Nrf2 up-regulation that leads to MDA suppression and antioxidant enzymes generation, such as superoxide dismutase and HO-1. Adenine damages the kidney through the elevation of ROS, while zinc stabilizes and protects the antioxidant enzymes to combat the imbalance and protect the kidney from oxidative damage [4].

This study showed the up-regulation of fibrotic genes in the renal tissue of adenine group. Also, western blot analysis proved the up-regulation of β-catenin proteins in adenine-fed rats. In contrast, ZnO-NPs and SPL group caused down-regulation of fibrotic genes, inflammatory genes, and up-regulation of antioxidant genes in renal tissue compared to each of SPL and ZnO-NPs groups. These results are in line with those of Diwan et al. [11], who established that adenine-fed rats have higher TGF-β, collagen, IL-6, and TNF-*α* expression while having lower Nrf2 and HO-1 expression.

Evidence suggests that TGF-β promotes the formation of reactive oxygen species, including fibrogenesis. MMP-9 and TIMP-1 production and activity are increased in CKD by the infiltration of inflammatory cells, the release of TNF-α, TGF-β, and ROS [23]. On the other hand, TGF-β activates both Smad-dependent and independent pathways causing several biological responses. When TGF-β1 binds to its receptor, TGF-β receptor type II (TβRII), the TβRI is activated and forms a heterodimer leading to Smad2 and Smad3 phosphorylation and binding to Smad4. After that, Smad2 and Smad3 are translocated to the nucleus leading to the expression of fibrogenic genes such as fibronectin and collagen [24]. Immunofluorescence examination of collagen III, and collagen IV represented the same findings. Diwan et al. [11] concluded that chronic inflammation was induced by a 0.25% adenine diet, which increased myofibroblast infiltration and macrophage, TNF-α, collagen, TGF-β, and α-SMA expression. Also, Oh et al. [25] revealed that Wnt/β-catenin signaling was up-regulated by an adenine diet by attaching Wnts to receptors on cell membrane, then Wnts dephosphorylate β-catenin. The cytoplasmic β-catenin is translocated to the nucleus, where it regulates Wnt7a target gene transcription. The up-regulation of β-catenin, Wnt1, Wnt2, and Wnt6 mRNA expression activated Wnt/β-catenin signaling. Similar findings were clarified by immunohistochemical investigation of β-catenin, TGF-β1, and α-SMA expressions.

Furthermore, western blot revealed a decrease or absence of β*-*catenin in the cytoplasm, but they were translocated into the nucleus, and Nrf2 nuclear translocation was elevated in ZnO-NPs and SPL group. A similar result was seen in an immunohistochemical examination of β-catenin, as β-catenin accumulates in the cytoplasm before translocation to the nucleus, where it regulates gene transcription [26]. According to Elseweidy et al. [8], SPL has anti-inflammatory properties that are demonstrated by inhibition of nuclear factor kappa B (NF-κB) and TNF-α down-regulation. In addition, the anti-fibrotic action of SPL is thought to be due to insulin growth factor 1 (IGF-1) down-regulation, which inhibits TGF-β1 expression and hence reduces kidney damage. Furthermore, Syngle and Verma [27] demonstrated that SPL decreased the pro-inflammatory cytokines (IL-1, TNF-α, and IL-6). SPL significantly attenuated Wnt/β-catenin signaling activation [28]. Furthermore, Rodri et al. [29] demonstrated a decrease in α-SMA-expressing cells, indicating fibrogenic inhibition in SPL-treated group. Immunohistochemical analysis of TGF-β1 and α-SMA expressions illustrated the same results. In addition, Rombouts et al. [30] showed that procollagen I and IV synthesis was inhibited by SPL, with a tendency to inhibit procollagen III. Similar findings were reported in the immunofluorescence investigation. Binding of the SPL to the mineralocorticoid and glucocorticoid receptors at high doses could be an explanation for decreased collagen production. The inhibition of collagen synthesis by dexamethasone or corticosterone activation of the glucocorticoid receptor in skin fibroblasts is well known. Also, Feria et al. [31] clarified that TGF-β1 deficiency inhibits the formation of extracellular matrix proteins like fibronectin, and collagen I, both of which are related to kidney damage. According to Li et al. [32], after one week of treatment with SPL, the levels of MMP-2, MMP-9, and TIMP-1 can be reduced. Meanwhile, Yuan et al. [33] explained that SPL up-regulated Nrf2 expression as the Nrf2 activation leads to transcriptional regulation of a variety of phase II detoxification and antioxidant enzymes, such as HO-1. These enzymes reduce oxidative stress in tissues and cells. The Nrf2 overexpression decreased renal TGF-β1, fibroblast cells, α-SMA, fibronectin and type 1 collagen. Furthermore, Yim et al. [34] stated that SPL up-regulated HO-1 in rat kidney.

According to Gulbahce-Mutlu et al. [35], ZnO-NPs reduced IL-6 in breast cancer in rats, indicating that ZnO-NPs have anti-inflammatory properties. Also, Bashandy et al. [9] established that ZnO-NPs inhibited the IL-6 and TNF-α. Since IL-6 deregulates antioxidant defenses, ZnO-NPs may reduce kidney injury by lowering IL-6 and lipid peroxidation levels. Additionally, ZnO-NPs could inhibit collagen bundles and α-SMA-positive cells in rats. Moreover, Alomari et al. [36] clarified that ZnO-NPs treatment decreased IL-6, TGF-β1, TNF-α, fibronectin, and collagen IV expression as illustrated in immunofluorescence examination. According to Guo et al. [37], ZnO-NPs inhibited MMP-9 and TGF-β1-induced fibroblast activation and epithelial differentiation. Furthermore, Sehsah et al. [38] reported that ZnO-NPs exposure resulted in an increase in *CAT, SOD, HO-1,* and *Nrf2* mRNA levels. In addition, Thomas et al. [39] explained that because ZnO-NPs reduced β-catenin expression, Wnt7a expression was reduced as well.

## Conclusion

Rat CKD was reduced by SPL combined with ZnO-NPs. The anti-inflammatory and anti-fibrotic properties of SPL and ZnO-NPs, as well as their antioxidant activities, can be used to treat adenine-induced CKD in rats. The inhibition of oxidative stress, the up-regulation of antioxidant genes (*Nrf2* and *HO-1*), the down-regulation of fibrotic genes (*TGF-β1*, *Wnt7a, β-catenin*, *fibronectin*, *collagen IV*, and *α-SMA*), and the down-regulation of inflammatory genes (*TNF-α* and *IL-6*) explain their effects. As a result, SPL combined with ZnO-NPs could be a potential CKD therapeutic approach.

## Data Availability

The datasets used and/or analyzed during the current study are available from the corresponding author on reasonable request.
